# Detection of Formaldehyde in Water: A Shape-Effect on the Plasmonic Sensing Properties of the Gold Nanoparticles

**DOI:** 10.3390/s120810309

**Published:** 2012-07-30

**Authors:** Sri Nengsih, Akrajas Ali Umar, Muhamad Mat Salleh, Munetaka Oyama

**Affiliations:** 1 Institute of Microengineering and Nanoelectronic (IMEN), Universiti Kebangsaan Malaysia, UKM Bangi 43600, Selangor, Malaysia; E-Mail: sri_nengsih85@yahoo.com; 2 Department of Materials Chemistry, Graduate School of Engineering, Kyoto University, Nishikyo-ku, Kyoto 615-8520, Japan; E-Mail: oyama.munetaka.4m@kyoto-u.ac.jp

**Keywords:** LSPR, gold nanoparticles, formaldehyde, plasmonic properties, optical sensor

## Abstract

The effect of morphology on the plasmonic sensing of the presence of formaldehyde in water by gold nanostructures has been investigated. The gold nanostructures with two different morphologies, namely spherical and rod, were prepared using a seed-mediated method. In typical results, it was found that the plasmonic properties of gold nanostructures were very sensitive to the presence of formaldehyde in their surrounding medium by showing the change in both the plasmonic peaks position and the intensity. Spherical nanoparticles (GNS), for example, indicated an increase in the sensitivity when the size was increased from 25 to 35 nm and dramatically decreased when the size was further increased. An *m* value, the ratio between plasmonic peak shift and refractive index change, as high as 36.5 nm/RIU (refractive index unit) was obtained so far. An expanded sensing mode to FD was obtained when gold nanostructures with nanorods morphology (GNR) were used because of the presence of two plasmonic modes for response probing. However, in the present study, effective plasmonic peak shift was not observed due to the intense plasmonic coupling of closely packed nanorod structures on the surface. Nevertheless, the present results at least provide a potential strategy for response enhancement via shape-effects. High performance plasmonic sensors could be obtained if controlled arrays of nanorods can be prepared on the surface.

## 1. Introduction

Gold nanoparticles have become a promising material for chemical sensing purposes in recent years due to their unique broad range of bio-compatibility, expanded catalytic behavior which is inherently related to the nanocrystals facet effect, and excellent chemical stability, as the basic parameters for interacting with analytes [1–3]. This material also exhibits peculiar optical properties [4–9] with the presence of surface plasmon resonance bands that are very sensitive to the changes in the dielectricity of the surrounding medium and their surface chemistry [1], making them a promising agent for the detection of analytes. On account of the quantum confinement effect, these properties can be finely tuned by simply changing their size and shape, expanding their performance in sensing applications. It is also well-known that the potential energy of the surface, which controls the surface chemistry (*i.e.*, surface reactions and the catalytic activity), of the gold nanocrystals depends on the available crystal facets. The surface energy on certain facet increases following the order of (111) < (100) < (110) [10]. On the basis of this fact, controlled but enhanced surface chemistry properties could be obtained if the gold nanocrystals are prepared in the form of certain unique morphologies in which they are bounded by a particular crystal plane, such as nanorods, nanocubes, *etc.*

Our group has been working on the optical sensing of variety of analytes including volatile organic compounds and agricultural product vapor detection using chromophoric compounds, such as phorpyrin, as the sensitive materials [11–16]. Photoluminescent quantum dots have also been used recently by us for a high-sensitivity detection of pesticides in water, which utilizes the unique photoluminescence quenching behavior of quantum dots upon the presence of pesticides in water [17]. Despite the fact that those materials exhibited excellent sensitivity by giving visible changes in the optical absorption and photoluminescence properties, they feature relatively low stability, in particular if used in liquid media. As an extended effort to obtain high-sensitivity but with a sensor system of suitable stability, we have employed gold nanoparticles as the sensitive agent in a plasmonic based detection of analyte molecules. Sensing responses towards glucose in water and gaseous molecules from VOC compounds have been obtained so far [18]. It was found that the SPR properties of gold nanoparticles were very sensitive to a small change on the dielectricity of their medium due to the presence of analytes or analyte molecules' adsorption onto the gold nanoparticles surface, which is indicated by the change in the plasmonic band intensity and the shift in the peak positions. As the fact of the plasmonic sensitivity to the change of the refractive index of the medium strongly depends on the morphology of the nanostructures [19], unique plasmonic responses should be obtained if nanostructures with different morphologies were used. In addition, due to the high biocompatibility of the gold nanostructure which is also influenced by the crystallographic plane [18,20,21], the molecules' adsorption onto the gold nanoparticles surface may also be considered to play an extended role in the change of their optical properties [10]. Thus, here we examined the morphology effect on the plasmonic properties of the gold nanostructures in the presence of formaldehyde using two different gold nanostructures, namely spheres and nanorods.

Formaldehyde (FD) is a colourless, tasteless and odorless chemical—at a certain low-concentration—that is widely used as preservative for biological specimens due to its active anti-microbiological properties [22,23]. Unfortunately, in many countries, its preservative function is extended to, to name a few, food and beverages, dairies, fresh sea-food and agricultural products and cosmetics [24]. Since the fact is that—at certain high-concentrations—it may be destructive to body organs and also cause serious effect to human health such as cancer and leukemia [22,23,25], there is a high demanded to control its presence in food products by developing a versatile detection method.

A number of methods have been available for detection of formaldehyde, such as fiber optical [26], electrochemical [27], ion chromatography, gas chromatography [28] and liquid chromatography methods [29] and high-performance liquid chromatography (HPLC) [30]. Despite the fact that these methods exhibit a sophisticated sensitivity if suitable conditions are fulfilled, the processes are complex, involving several suitable sample pre-treatments, such as heating or adding specific reagent to the specimens. Thus, in an effort to develop a simple detection technique but with appropriate sensitivity characteristics, we investigated the optical responses of gold nanostructures with two different morphologies, namely spherical and nanoparticles and nanorods, in the presence of FD. In a typical result, it was found that the optical properties of the gold nanostructures of spherical and rod morphology responded differently to the presence of FD, whereby the optical absorbance of the nanostructures increased independent of the wavelength and decreased with the increasing FD concentration for spherical nanoparticles and nanorods, respectively. Owing to the unique optical responses of these nanostructures to the presence of FD, the present approach should find extensive use in the detection of FD in food products. The mechanism for the responses of gold nanostructures related to their morphology is discussed.

## 2. Experimental Section

### 2.1. Growth of Gold Nanoparticles on the Surface

#### 2.1.1. Spherical Nanoparticle Preparation

Spherical gold nanoparticles were prepared on a quartz substrate surface using our previously reported method [7,8], namely seed-mediated growth. This method comprises a simple two-step processes, namely seeding and growth processes. Briefly, the seeding process was carried out by simply immersing a clean quartz substrate (Fuzhou WTS Photonics Co. Ltd., Fuzhou, China) into a 20 mL aqueous solution that contains a mixture of 0.5 mL of 0.01 M HAuCl_4_·3H_2_O (Aldrich, St. Louis, MO, USA), 0.5 mL of 0.01 M trisodium citrate (WAKO Chemical, Osaka, Japan). The substrate sample was kept for 30 min in the solution to facilitate gold ions adsorption onto the substrate surface and after that a 0.5 mL of ice-cold 0.1 M NaBH_4_ (WAKO Chemical) was then added to the solution to induce the formation of gold nanoseeds on the surface. The addition of NaBH_4_ into the solution changed the solution appearance from colorless to red, an indication of nanoseed formation. The substrate was further kept in the solution for another 1 h. After that, the sample was taken out, rinsed with pure water and dried with a flow of nitrogen gas. Using this approach, gold nanoseeds with sizes ranging from 2 to 5 nm could be obtained on the surface.

The nanoseeds on the substrate surface were then grown by immersing the substrate into a solution that contains 20 mL of 0.1 M CTAB (Amresco, Solon, OH, USA), 0.1 mL of 0.1 M ascorbic acid (WAKO Chemical) and 0.5 mL of 0.01 M HAuCl_4_·3H_2_O (Aldrich). A reddish color on the substrate may be observed within seconds after the substrate immersion into this solution indicating the formation of the gold nanoparticles. Gold nanoparticles with different size could be realized on the surface by varying the immersion time in the solution. Next, the substrate was removed and then rinsed thoroughly with a copious amount of pure water and dried with a stream of nitrogen gas. Finally, the sample was annealed at 200 °C in air for 1 h to remove any organic residues on the surface.

#### 2.1.2. Nanorod Preparation

The gold nanorods in solution were prepared following the technique developed by Nikoobakht and El-Sayed [31] with several modifications [32]. In this technique, two solutions were prepared for the growth of nanorods, namely seed solution and growth solution. Firstly, gold seed solution was prepared by adding 0.25 mL of 0.005 M HAuCl_4_·3H_2_O into 5 mL of 0.2 M CTAB and shaken for 1 min to mix the solution. Next, freshly prepared 600 μL ice-cold 0.01M NaBH_4_ solution was added to the solution. The color of the solution changes from light yellow to brownish after adding NaBH_4_ indicating the formation of nanoseeds in the solution. Secondly, the growth solution was prepared by adding 0.1 mL of 0.1 M L-ascorbic acid into a solution that contain 5 mL of 0.2 M CTAB, 0.1 mL of 0.005 M HAuCl_4_ and 0.15 mL of 0.004 M AgNO_3_. After that, 0.1 mL of the nanoseeds solution was injected into the growth solution followed with a rigorously shaking for 10 s to mix the reagents. Then, the reaction was left undisturbed in a controlled temperature water bath at 28 °C. After 2 h, the reaction was stopped and the nanostructures precipitate was collected and washed with pure water by centrifugation. The precipitate was finally dispersed in ethanol. The nanorods were then deposited onto a quartz substrate by a spin-coating method at 1,000 rpm for 40 s. The sample was kept in a dry box before further use in sensing applications.

### 2.2. Characterisations

The morphology of the as-prepared gold nanostructures on the substrate surface was studied using a field emission scanning electron microscope (FESEM) machine model ZEISS SUPRA 55VP that was operated at an acceleration voltage of 3 kV. The optical property of the nanostructures was characterized using a double beam Perkin Elmer UV/VIS/NIR spectrophotometer model Lambda 900.

### 2.3. The Optical Responses Study

**Figure 1.**
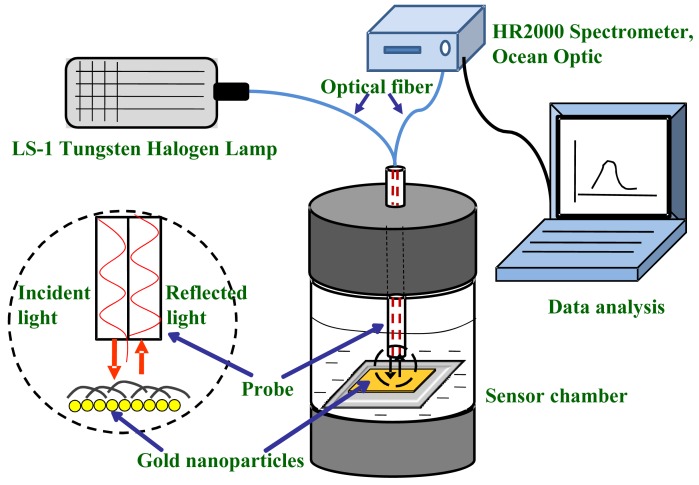
Sensing system set up utilizing fiber optic and gold nanoparticles.

A sensor system as shown in Figure 1 was set up for investigating the optical response of the gold nanoparticles to the presence of formaldehyde in solution. The setup consists of one chamber for liquid samples, a light source (LS-1 tungsten halogen lamp), two arm fiber optical probe system and an HR-2000 Ocean Optics spectrometer. The light source, fiber, and the spectrometer were purchased from Ocean Optic Inc. (Dunedin, FL, USA).

To examine the response of gold nanoparticles to the presence of formaldehyde, the substrate containing gold nanoparticles was firstly immersed into a water media and laid flat at the bottom of the water container around 1 mm from the surface. A duplex fiber probe (a probe with two fiber-arms) was positioned approximately 0.5 mm above and normal to the substrate surface. In other words, the probe-end was also immersed into the water to avoid unwanted reflected light from the water surface. The responses were studied by transmitting the light from the source into one of the fiber arms as shown in the figure, reaching the gold nanoparticles sample at the other end (probe end). Some light was scattered back to the probe and the rest was transmitted through the substrate, reaching the rough Teflon liquid container base and then reflected back to the probe. The scattered light was collected by a probe and transmitted to the optical analyzer using another arm of the fiber. The scattered light that comes from the bottom of the liquid container as well as the contribution of medium refractive change to the absorption spectrum profile were discarded by recording the reference spectrum of the light using a blank quartz substrate. Therefore, the recorded light spectrum by the spectrometer solely came from the scattered light by the gold nanoparticles. The plasmonic sensitivity property of the gold nanoparticles to this chemical were studied by obtaining the optical absorption of the gold nanoparticles in the presence of formaldehyde with several concentrations, ranging from 3 to 37%.

## 3. Results and Discussion

### 3.1. Spherical Nanoparticles and Nanorods

**Figure 2.**
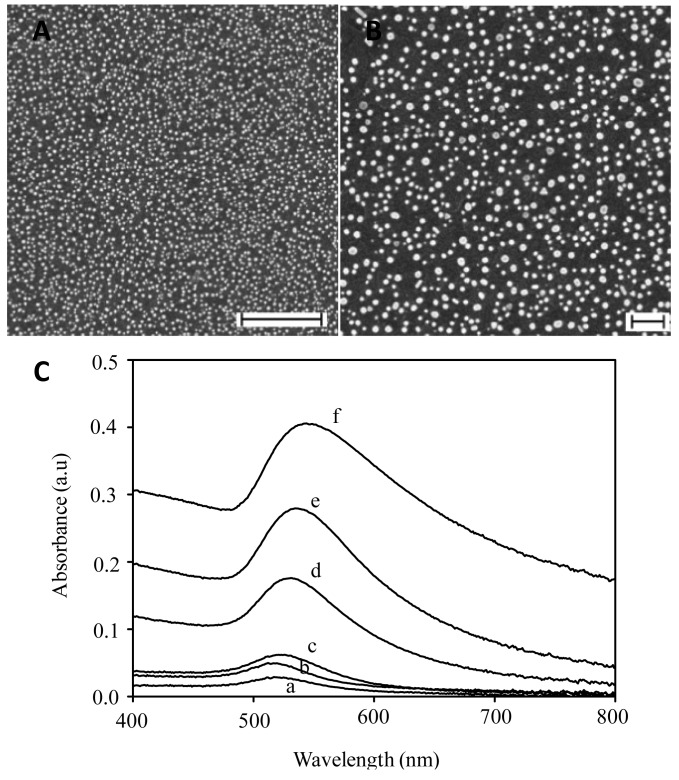
(**A** and **B**) FESEM images of spherical gold nanoparticles that were grown on the surface for 2 h in the growth solution; (**C**) The optical absorption spectrum of gold nanoparticles on the surface that was grown for different growth time, namely (a) 0.5; (b) 1; (c) 2; (d) 4; (e) 8 and (f) 16 h. Scale bars in A and B are 100 nm.

**Figure 3.**
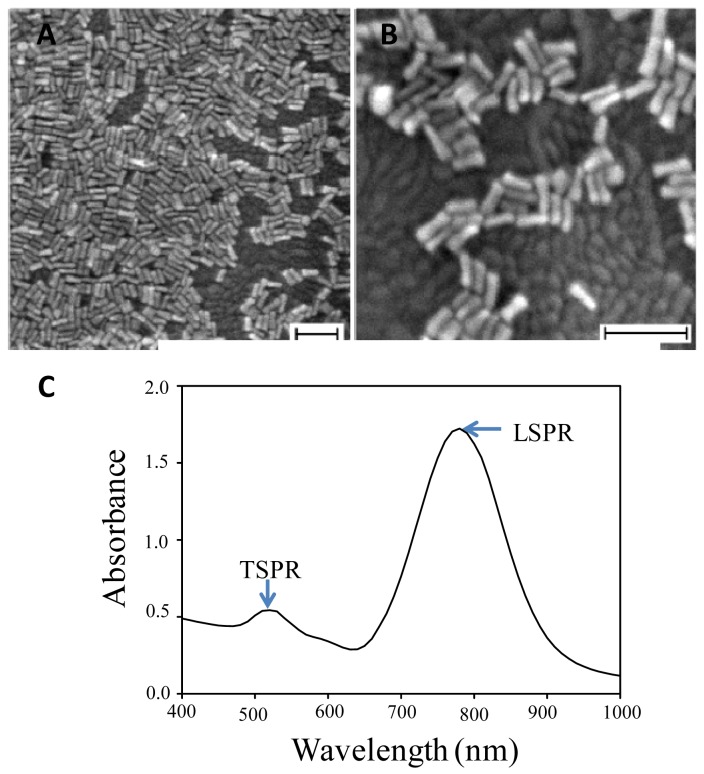
(**A,B**) Typical FESEM image of gold nanorods attached on the substrate surface; (**C**) Corresponding optical absorption spectrum of gold nanorods in solution phase showing two clear plasmonic modes, transverse and longitudinal for shorter and longer band, respectively. Scale bars in A and B are 100 nm.

**Figure 4.**
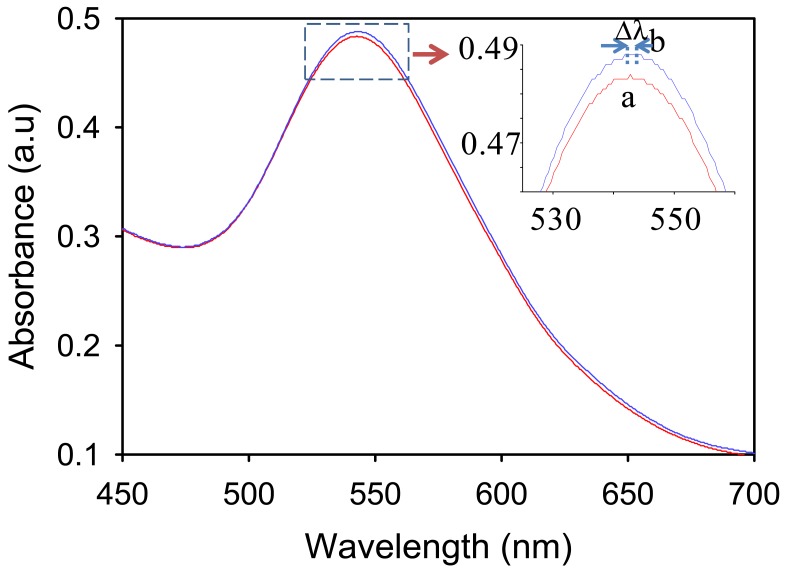
Optical absortion spectra of GNS in water (a) and in the presence of 10% formaldehyde (b). Inset graph indicates the change in the spectra involves absorbance change and peak shifting.

We have effectively prepared gold nanoparticles with two different morphologies in this work, namely spherical and nanorods, using the approaches which are described in the Experimental section. The FESEM image and their related absorption spectra are shown in Figures 2–4.

The spherical nanoparticles (see Figure 2), were found to exhibit a relatively perfect spherical morphology with diameters in the range of 30 to 35 nm approximately. As evident from the figure, the nanoparticles were distributed evenly on the entirety of the surface without the presence of agglomeration amongst the nanoparticles. By using an image analysis tool, the surface that is covered by the nanoparticles can be estimated to be as high as ca. 35%. This condition might preserve the plasmonic properties of the individual nanoparticles due to their localized nature and makes them suitable for localized-surface plasmon resonance sensing purposes. The nanoparticles' diameters could be further varied from 25 to 47 nm by simply varying the growth time from 0.5 min to 16 h in the reaction. Figure 2B shows related absorption spectra of the spherical gold nanoparticles. As can be seen from the figure, a single absorption band centering at 517 nm, which is the surface plasmon resonance of spherical gold nanoparticles, characterized the optical absorption spectrum. The center of the band is red-shifted with the increasing nanoparticle diameter, namely from 517 nm for nanoparticles with diameter of 25 nm to 544 nm for a diameter of 47 nm. This is the unique size-optical properties dependence of the gold nanoparticles, whose optical properties strongly depend on the size as the result of the quantum effect, which is promising for optical sensing sensitivity modification based on the surface plasmon resonance property. As has been mentioned earlier, modification of nanoparticles size will directly modified all the properties of the nanoparticles, in particular the surface chemistry of the nanoparticles, a key factor that influences the short-range interaction of analytes and nanoparticles. Since the optical properties of the nanoparticles also depend on the size and the surface chemistry as well as the dielectricity of the medium, unique plasmonic properties due to the presence of the analytes will be obtained when the nanoparticles size is varied.

Figure 3 shows the FESEM image of the nanorods on the substrate surface prepared in this study. As can be seen from the image, nanorods have been effectively attached on the surface covering the majority of the surface [see Figure 3(A)].

On the basis of the low-resolution FESEM image, we have made an image analysis and found that the surface covered by the nanorods can be estimated to be ca. 60%. Further analysis on the morphology of the nanorods revealed that they had relatively homogenous lengths, but exhibit a slight variation in their diameter, in which is high at the nanorod ends and lower in the center of the body of the nanorods, making them resemble bone-like structures. In a typical process, nanorods with diameter and length of ca. 10 and 40 nm or aspect ratio of ca. 4 are the characteristic. As also evident in the figure, the nanorods tend to arrange side by side to each other, instead of being localised on the surface. This could probably be associated with the following effects: (i) the effect of the solvent drying process; and (ii) the effect of the surface energy minimalisation process amongst the nanorods, which is mostly covered by high-energy crystal planes, namely (110). It is true that the latter process may produce a continuous film of GNR on the surface and as the result the absorption spectrum will lose its localized character. However, this did not occur (this will be discussed later) because the surfactant molecules that cover the GNR hinder the GNR from being in contact each other. Thus, the localized character of the plasmonic spectrum is preserved.

Figure 3C shows a typical optical absorption spectrum of gold nanorods in the solution. As revealed in the figure, in contrast to those shown for spherical nanoparticles, the spectrum exhibits the presence of two major absorption bands that are centered at 528 and 650 nm. These two bands are attributed to the transverse- and longitudinal-surface plasmon resonance for the shorter and the longer wavelength, respectively. Such a unique optical feature demonstrated by the nanorods might probably expand their functionality in applications because of the presence of two wavelength domains (*i.e.*, transverse and longitudinal) which presumably have different interplay with the ambient. Thus, they produce unique different sensitivity to the changes of the surrounding medium properties.

### 3.2. Plasmonic Sensing Responses

#### 3.2.1. Spherical Nanoparticles

In our previous study, we have examined the plasmonic responses of the spherical gold nanoparticles (GNS) to the presence of formaldehyde (FD) in water and the effect of their size on the sensing sensitivity [33]. It has been reported by us that the sensitivity was found to increase with the increasing size, from 25 nm, and the optimum was at the size of ca. 35 nm. Surprisingly, the response then dramatically decreased when the GNS size was further increased, for example 47 nm. This result and the phenomenon of unique size-response relationship obtained in this study are likely normal and agree with those reported by Nath and Chilkoti earlier [34]. This can be easily related to the nature of the bulk sensing volume of the gold nanoparticles (*i.e.*, attributed to the electromagnetic field penetration depth of the nanoparticles), which is dependent on the size and morphology of the nanoparticles, that increases with the increasing size, but is optimized at a particular dimension [19]. Thus, we obtain variable refractive index sensitivity when the nanoparticle's size is varied. Short-range interactions between GNS and FD that lead to “local refractive index change” (the volume in the vicinity of the GNS) [34], which also depends on the GNS size, might also be considered to play a certain role in such plasmonic response properties. However, in this case, it could be relatively small and it may quickly reach a saturated-state for the case involving the adsorption process. Therefore, bulk solution refractive index changes become the major factor for the optical properties change. On the basis of our earlier study, we recognized that the GNS of size ca. 35 nm, which is prepared by growing the sample for 3 h, was the size that give the optimum plasmonic responses. Therefore, in the present study, we only concentrated on using this optimized-size as the basis of the study of the effect of morphology on the plasmonic sensing sensitivity.

Figure 4 shows typical absorption spectra of the GNS of size ca. 35 nm in water and in the presence of 10% aqueous solution of FD. Frankly, when looking at the figure, no clear differences were observed on the spectra when the GNS were exposed to the FD. However, surprisingly, upon a closer look on the spectra, a relatively large spectral difference amongst the two samples was observed. As can be seen from the spectra at the inset of Figure 4, the change involves the increase in the absorbance of the GNS, independent of the wavelength, and effective red-shifting of the peak of the plasmonic band. On the basis of the spectra, the absorbance change and the peak shifting as high as ca. 0.05 and 3 nm respectively were obtained.

**Figure 5.**
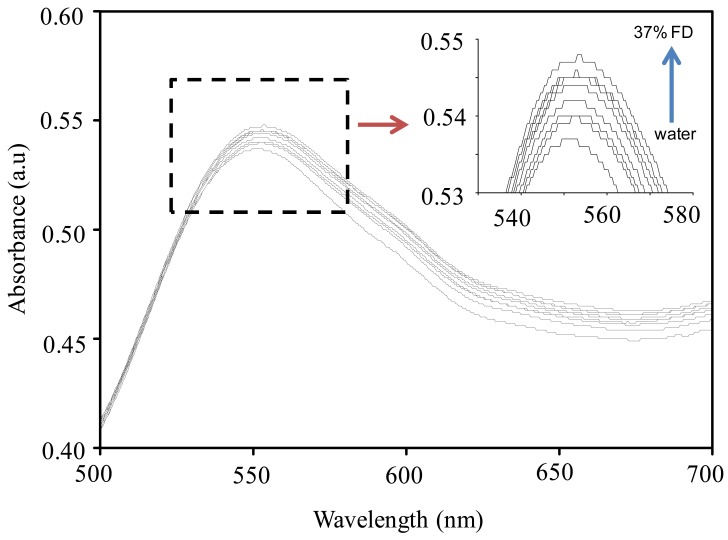
Absorption spectra of GNS in the presence of several concentration of FD. The absorbance of the GNS increase and the plasmonic peaks red-shift with the increasing of the FD concentration.

We further examined the plasmonic response of the GNS to the presence of several concentrations of FD. The results are shown in Figure 5. As revealed in Figure 4, the optical absorption of the GNS gradually modified upon the increase of FD concentration that indicated by effective increase in the absorbance as well as red-shifting in the plasmonic peaks as the increasing of FD concentration. From the spectra, it can be found that the introduction of FD at concentrations as low as 3% has effectively changed both the intensity of the absorbance and the plasmonic peaks' position (red-shift). This change was found to further improve with the increase in FD concentration up to 37% (also called 100% formalin), reflecting excellent response linearity.

From the figure, it is also found that the presence of FD in the GNS vicinity had caused an increase in the effective area under the curve. Since this area is related to the concentration of the chemical in the medium, which is also related to the refractive index of the medium, these results confirm the exceptionally high sensitivity of GNS to the medium's refractive index change, affirming the nature of the plasmonic sensing of FD is via long-range plasmonic response (bulk solution refractive index). One may say that if plasmonic responses are simply due to the changes in the refractive index of the medium in the presence of FD instead of interaction with the GNS, the GNS system might have a poor selectivity property toward different analytes, which thus limits their use in applications. However, as we mentioned earlier, the plasmonic response also involved short-range interactions with analytes, such as via adsorption or catalytic reactions, so the GNS should also have a probability to produce unique responses characteristic of long-range interactions, which are specific to a certain analyte system due to the quantum effects in this length-scale regime. Thus, selectivity can be obtained. Therefore, we were convinced that the plasmonic responses of the GNS toward different analytes should also be different. A detailed analysis on the sensing sensitivity of GNS towards different analyte system is being pursued and will be reported in another publication.

**Figure 6.**
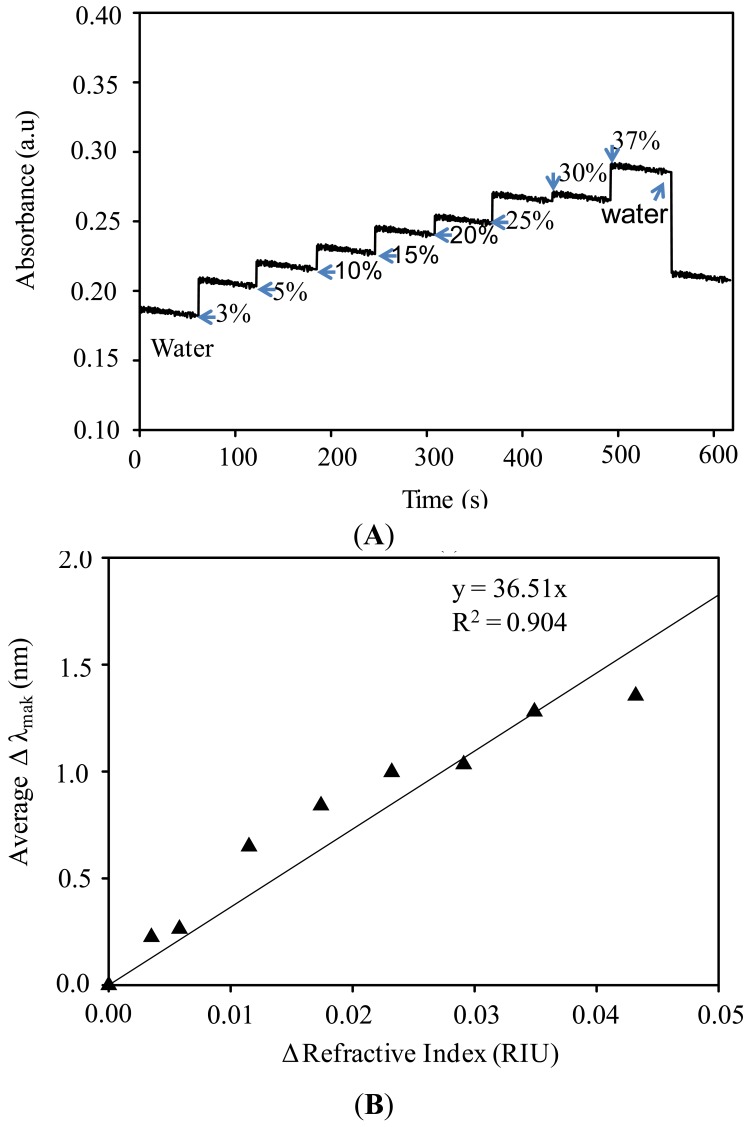
(**A**) Dynamic response of GNS with size of ca. 35 nm under change of FD concentrations that measured at the plasmonic peak namely 552 nm; (**B**) Plot of average plasmonic peaks shift *versus* medium refractive index change.

Figure 6A shows the corresponding dynamic response of the GNS measured at the peak of the plasmonic band, namely 552 nm, under the introduction of several concentrations of FD. As can be seen from the figure, it is confirmed that the GNS exhibit excellent response linearity with the variation of FD concentration. It can also be noted here that the plasmonic response of the GNS was relatively quick with a typical response time, the time to reach the maximum change in the absorbance, of ca. 20 s. The response was found to fully recover back to the original condition when FD was removed from the vicinity of the GNS system. The recovery time was calculated to be ca. 25 s. These results reveal that the plasmonic property of the GNS is very sensitive to the FD, on account of their high-sensitivity to the change of the refractive index of the medium.

We then examined the nature of GNS plasmonic sensitivity to the change in the refractive index of the medium by interpolating the plasmonic band peak shift in the change in the refractive index of the medium. The plasmonic band peak shift was calculated directly from the spectra by comparing the peak position of the plasmonic band with the original position. Meanwhile, the refractive indexes of the medium under certain FD concentrations were calculated using a Lorentz-Lorenz approximation [35]. The result is shown in Figure 6B. As can be seen from the figure, in good agreement with the results as shown in Figures 4 and 5, the plasmonic peaks shift is linearly increased with the increase of the refractive index of the medium, confirming the excellent linearity of the plasmonic responses of the GNS to the FD sample. By using a linear regression method, plasmonic sensitivity can be calculated to be as high as ca. 36.5 nm/RIU (refractive index unit). The change in the plasmonic peak of the GNS in the presence of FD can be directly understood as the result of a strong dependence of plasmonic of the GNS on the dielectricity of the surrounding medium [36]. In this case, the medium was changed from water to FD. Since the dielectricity of the medium can be represented by the value of the refractive index, the change of water to, for example, 10% FD commensurate to the change of the refractive index as high as 0.005. This shifted the plasmonic peaks and at the same time increased the absorbance as mentioned earlier.

#### 3.2.2. Nanorods

It is a unique feature that the gold nanorod (GNR) exhibits two characteristic surface plasmon resonances in the optical absorption spectrum, namely transverse and longitudinal mode. As mentioned previously, the transverse surface plasmon resonance (TSPR) mode appears at a relatively higher electromagnetic energy than the longitudinal surface plasmon resonance (LSPR) mode. As has been hypothesized earlier, such unique properties might produce special sensing characteristics resulting from the unique plasmon-analyte interaction at these two SPR energies.

**Figure 7.**
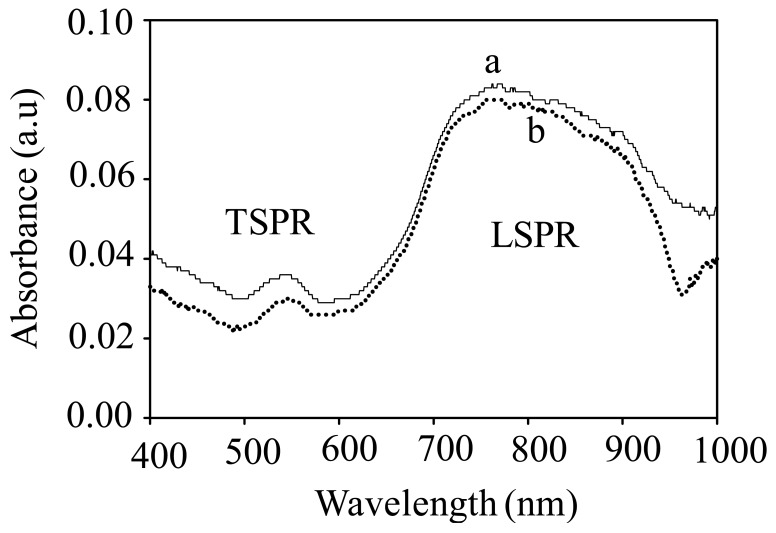
Optical absorption spectra of GNR on the substrate surface in water (a) and in the presence of 10% formaldehyde in water (b).

Figure 7 shows typical optical absorption spectra of the GNR on the surface in water and in the presence of 10% FD. One point to be noted here is that the obtained spectrum of GNR is not similar to and distorted from that observed in solution (see Figure 3C), in particular at the longitudinal part of the SPR, which exhibits a broader band. This might be due to the effect of intense aggregation amongst the nanorods on the surface, as confirmed by the FESEM image shown in Figure 3. As can be seen from the figure, the absorbance of the GNR independent of the wavelength effectively decreases when the FD is introduced into the surroundings of the GNR, reflecting excellent sensitivity of GNR to the presence of FD in solution. However, as can be seen from the curve, at wavelengths above 950 nm, an absorption tail was observed when the FD was introduced. This could be probably due to an effective interaction of FD and the plasmonic field at this wavelength regime. One point to be noted here is that this response is actually in contrast to those shown by the GNS, in which the absorbance was increased when the medium was changed from water to the FD. Unfortunately, different from those obtained in GNS, in the present system, plasmonic peak shifting at both SPR modes was not obtained. This could be due to the effect of the nature of the optical property of the GNR on the surface, which shows a distorted SPR profile compared to those obtained in solution, a result of GNR-GNR plasmonic coupling in such a close-packed GNR arrangement on the surface. Thus, the shift in the plasmonic absorption peaks due to the change in the refractive index of the medium could not be clearly observed in the spectra. It is true that the current results might not be the typical responses of nanorods to the FD due to the fact the nanorods are not completely localized on the surface. Nevertheless, the present result at least provide promising evidence that the nature of the analyte interaction with nanoparticles is strongly influenced by the shape of nanoparticles. If controlled-arrangement of nanorods on the surface is achieved, enhanced plasmonic sensing will be obtained as a result of the presence of strong localized effects on the SPR of the nanorod. This effort is underway.

**Figure 8.**
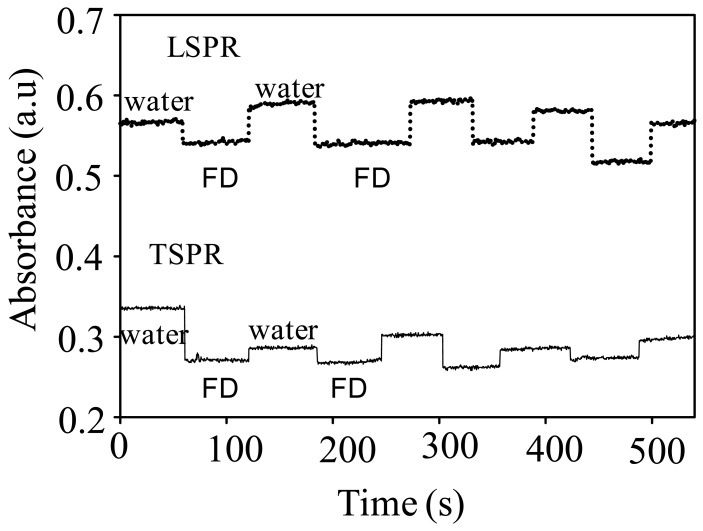
Plasmonic responses of GNR upon the presence and absence of FD (10%) measured at the TSPR and LSPR peaks' wavelength.

Figure 8 shows the responses of GNR to the presence of 10% FD in water that were measured at the peak wavelength of TSPR and LSPR. As has been revealed in the figure, the GNRs exhibits an excellent sensitivity to the presence of the FD in their surroundings and recovered back to their original condition when FD was replaced by pure water. It can also be seen that the responses of the GNR measured at the LSPR peaks shows a relatively higher response than those measured at the TSPR, indicated by the decrease in the absorbance upon exposure to the FD samples. It can also be noted that despite the fact of the fluctuation in the reponses upon multiple application of FD samples to the sensor system, it can be concluded that the GNR exhibited relatively high repeatability and stability properties. One important point should be noted from these results—the use of the GNR may expand the measurement due to the presence of two measurement modes, namely TSPR and LSPR. Although relatively similar responses between the TSPR and LSPR to the current analyte system are seen, the present fact more or less has provided a potential strategy for expanding the sensing properties of the metallic nanostructures system. The response of both mode of operations might be different if the GNR system was used to detect other analyte system, such as bioorganic molecules, *etc.* This enables the extensive use of GNR compared to the GNS system in plasmonic sensing applications in future.

**Figure 9.**
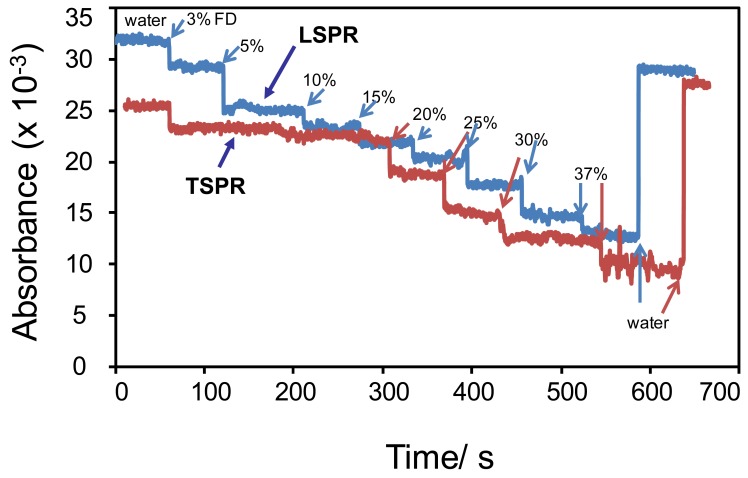
Dynamic response of GNR under variation of FD concentrations measured at the plasmonic peak, namely 552 nm.

Figure 9 shows the responses of the GNR under various concentration of FD that were measured at both plasmonic peaks. As can be seen from the figure, generally the GNR shows good response characteristics as measured at the two plasmonic peaks, in particular at the LSPR mode, by showing a linear decrease in the absorbance with the increasing FD concentration. The response can be very well-returned to the original position when the FD were removed from the sensor chamber. However, when measured at the TSPR mode, judging from the absorbance change, the GNR shows a lower sensitivity if compared to the measurement performed at the LSPR mode as the presence of small changes in the absorption when the FD concentration was increased in the chamber. It was also found that, in this TSPR mode, the GNR response exhibits an intense fluctuation when high FD concentrations are used. Actually, this phenomenon is quite abnormal since the gold nanostructure is very sensitive to the change in the refractive index of the medium as demonstrated in Figure 6 for the case of GNS. This could be probably due to the effect of strong plasmonic coupling between the GNR as the result of closely packed-structure on the surface. This probably decreases the sensitivity as well as the stability of the responses to the variation of FD concentration in the surrounding medium. Thus, the response fluctuates. Despite the fact the present results for GNR indicate a relatively low stability and performance if related to the unique morphology of the gold nanostructures, we hypothesize that if the GNR can be prepared on the surface with ordered-arrangement, the plasmonic detection should be enhanced. Therefore, the effort to prepare the GNR with ordered-arrangement on the surface is being pursued.

## 4. Conclusions

The plasmonic responses of two different gold nanostructures morphologies embedded on a quartz substrate, namely gold nanoparticles (GNS) and gold nanorods (GNR), to the presence of formaldehyde in water has been investigated. For the case of GNS, it was found that their plasmonic responses exhibited high-sensitivity to a small change in the refractive index of the medium due to the presence of formaldehyde (FD). The plasmonic responses were shown by increasing the intensity as well as the shift in the peak position of the plasmonic band upon the presence of FD in the aqueous medium. The responses were found to linearly increase with the increasing concentration of FD. By considering the peak shift and the refractive index value of the medium, a sensitivity factor *m*—the ratio of peaks shift and the refractive index value—as high as 36.5 nm/RIU was obtained for the GNS system. The plasmonic responses of gold nanostructures on the FD when their morphology was changed to gold nanorods (GNR) have also been obtained. Unlike in the GNS system, the GNR exhibited the presence of two plasmonic characters, *i.e.*, transverse and longitudinal surface plasmon resonance modes that showed unique response properties to the presence of FD. In a typical process, it was found that the intensity of the two plasmonic peaks significantly decreased when the FD sample was introduced into the surroundings of the GNR system. These results were in total contrast to those obtained for GNS. Despite the fact that no significant shift on the peak position of the two plasmonic effects were observed, the present result has provided a clear fact about the strong effect of morphology on the plasmonic responses of the gold nanostructures to the presence of FD. By optimizing the arrangement of GNR on the surface that minimizes the plasmonic coupling effect, enhanced plasmonic sensitivity will be obtained in the future. The effort for achieving appropriate GNR arrangement on the substrate surface is being pursued at the moment and their sensing characteristic to certain analytes will be reported in a subsequent publication.
